# Primary Stromal Sarcoma of Breast: A Rare Entity

**Published:** 2017-01-02

**Authors:** Sanjay Kumar, Jyoti Sharma, Megha Ralli, Gurpreet Singh, Sonu Kalyan, Rajeev Sen

**Affiliations:** *Dept. of Pathology, PGIMS, Rohtak (Haryana), India*

**Keywords:** Angiosarcoma, Bone, Vascular Tumor

## Abstract

Primary soft tissue sarcomas of the breast constitute less than 5% of all soft tissue sarcomas and less than 1% of malignant breast cancers. The rarity of this tumor limits most studies to small retrospective case reviews and case reports. Primary breast sarcomas are locally aggressive tumors as evidenced by the high rate of local recurrence when excisional surgery is performed. A contemporary multidisciplinary approach to therapy including surgery, radiation, and chemotherapy is advocated. Herein, we report a case of 45-yr-old female, who presented with a large ulcerated breast mass and was diagnosed as carcinoma breast on fine needle aspiration. Modified radical masectomy was performed and was diagonsed with primary breast stromal sarcoma on histopathology, which is a rare entity.

## Introduction

The breast is the place of metastatic sarcomas as well sarcomas secondary to radiotherapy (1). Primary breast sarcoma is a rare entity and occurs in less than 1% of women with breast malignancy, first described in 1887 (2). These sarcomas arise from the mesenchymal tissue of the mammary gland (3) and sarcomas arising from the skin, muscle, and adjacent bone are excluded (4). The etiology of primary breast sarcoma is largely unknown (4). 

Surgical resection is first line of treatment for these lesions and axillary lymph node dissection is not indicated in absence of palpable axillary lymph nodes. An adequate resection margin is most important factor determining long-term survival (5). Primary sarcomas of the breast are rare, malignant tumors and since only case reports or small series are available in literature, this entity is not well-understood (6). 

We report a case of 45-year-old female in which histopathological examination following mastectomy confirmed high-grade primary stromal breast sarcoma. Patient is on regular follow up six monthly.

## Case report

A 45-year-old female came to Surgery Outpatient Department with a complaint of progressively increasing ulcerated mass in right breast. Informed consent was taken from the patient. There was no family history of breast cancer. In addition, there was no history of breast trauma or any radiation exposure. On examination, there was a large ulcerated mass involving right breast. X-Ray chest and abdominal scan were normal. Her hemoglobin was 7.5 gm/dl. Fine needle aspiration performed was suggestive of carcinoma breast. Further, Modified radical mastectomy (MRM) was performed and the specimen was sent for histopathological examination. 

**Fig. 1:**
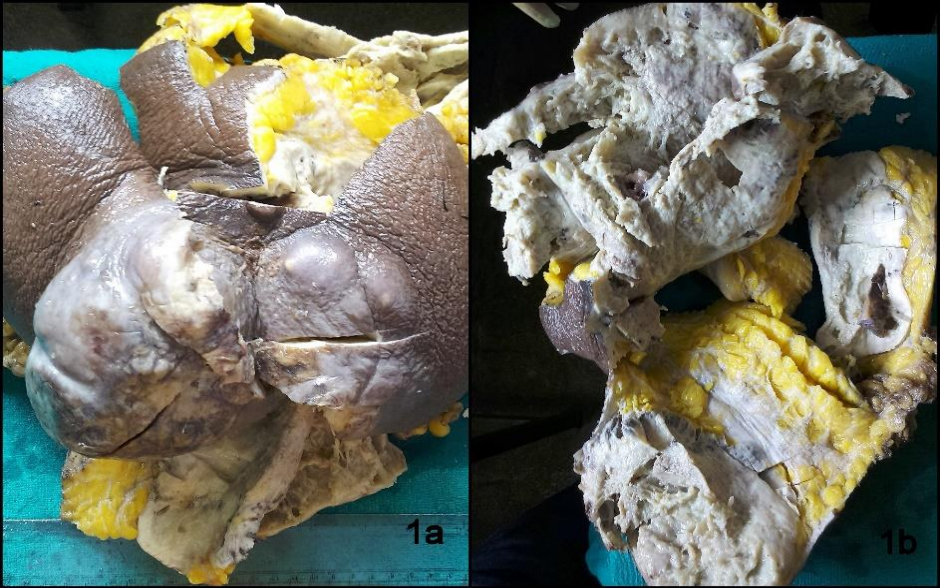
(a) Mastectomy specimen showing ulcerated and lobulated growth, (b) Cut surface is grey-white, solid with necrotic and cystic areas

Grossly, we received an MRM specimen measuring 24x15x13 cm along with skin flap measuring 14x9x6 cm. The skin surface showed a large grey-white lobulated and ulcerated growth (Fig. 1a). On serial sectioning, an ill-defined grey-white growth identified measuring approximately 18x15x12 cm that was involving the skin and appeared approximately 0.8 cm away from resected base (Fig. 1b). The cut section was solid, grey-white along with necrotic and few cystic areas. Totally, 10 lymph nodes were isolated from the specimen. 

**Fig. 2:**
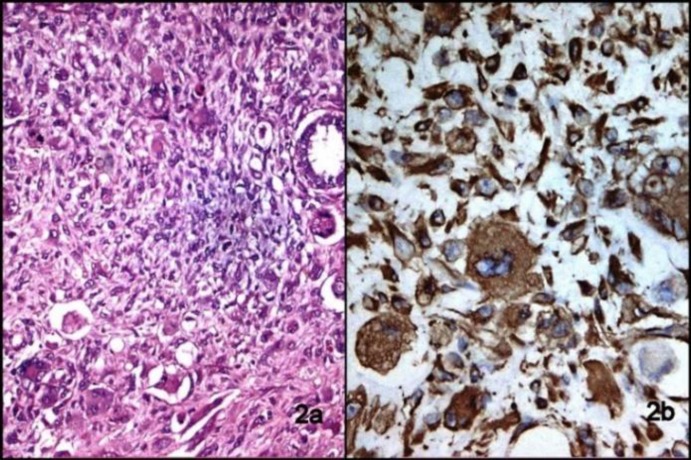
(a) Photomicrograph showing spindle tumor cell with variable size, round to oval nuclei, inconspicuous nucleoli and moderate eosinophilic cytoplasm, Multinucleate and bizarre forms also seen (H&E, 100X), (b) Tumor cells showing vimentin positivity (IHC, 200X

**Fig. 3:**
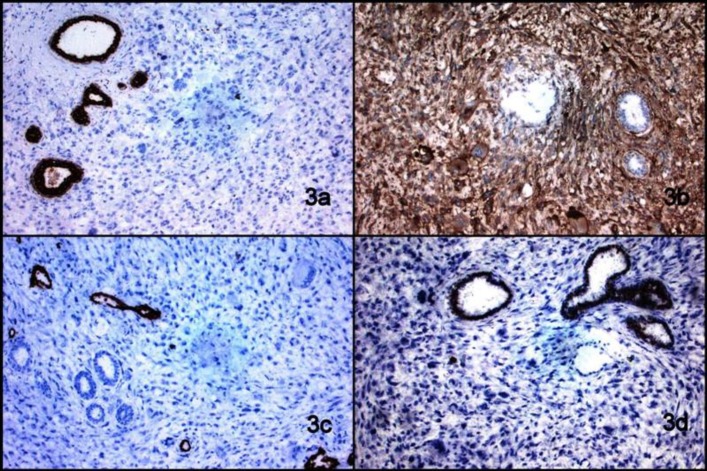
a) Photomicrograph showing cytokeratin positivity in benign epithelial cells (IHC, 100X), (b) Photomicrograph showing CD10 positivity in tumor cells (IHC,100x), (c) Photomicrograph showing CD34 positivity in endothelial cells of vessel wall (IHC,100X), (d) Photomicrograph showing ER positivity in benign epithelial cells (IHC,100X

Microscopic examination revealed numerous haphazardly arranged spindle cells with variation in cellular size along with few benign appearing glands entrapped in between. The cells had round to oval hyperchromatic nuclei with inconspicuous nucleoli and moderate amount of eosinophilic cytoplasm (Fig. 2a). Multinucleated and bizarre forms were also evident. Mitotic count was 4-5/10hpf. Possibility of soft tissue sarcoma was kept. Thorough sampling of the specimen was done to rule out the possibility of cystosarcoma phylloides. Further, immunohistochemical panel was applied including vimentin, cytokeratin (CK), Myogenin, desmin, SMA, S100, CD68, CD34, CD10, ER, PR, and Her2/neu. Vimentin and CD10 were positive in tumor cells; however, CK and ER were positive in benign epithelial cells (Fig. 2b, Fig. 3). CD34 was positive in endothelial cells of vessels. Desmin, Myogenin, SMA, CD68, S100, PR, and Her2/neu were negative. Lymph nodes isolated showed no evidence of metastatic deposits from tumor. A Final diagnosis of primary stromal breast sarcoma was made.

## Discussion

Breast cancer is the most common cancer of female group (1). Most invasive breast neoplasms are epithelial tumours and mesenchymal breast neoplasms are rare (7). Primary sarcomas of the breast are extremely rare with few hundred cases described in literature until today (1). Primary breast sarcomas occur over a wide range but most occur in women in their fifth or sixth decade of life (8). Male cases usually represent less than 5% of primary breast sarcomas (3). 

Sarcomas can occur anywhere in the human body. About 40% of sarcomas occur in the extremities with two-thirds accounted in the lower limb and one-third in the upper limb (7). Primary breast sarcomas are classified according to the criteria applied to the soft tissue counterparts (5). Most cases of breast sarcoma are secondary to radiotherapy due to increasing use of breast radiation after breast conserving treatment (7). PBS arise from the mesenchymal tissue of the mammary gland and the pathologic definition of this entity is clear (6). The variety of cells present in this tissue such as fat cells, muscle cells, or endothelial cells explains the heterogeneity of the histological types (5). Histological description was used by cell of origin of neoplasm. The most common subtypes are malignant fibrohistiocytoma, fibrosarcoma, angiosarcoma, and spindle cell sarcoma. Other subtypes are liposarcoma, leiyomyosarcoma, rhabdomyosarcoma, osteosarcoma, synovial sarcoma, neurosarcoma, stromal sarcoma, chondrosarcoma (1).

A group of mammary sarcomas is referred to as stromal sarcomas (5). Callery et al. used this term for tumors arising from specialized hormone sensitive stroma (9). However, now stromal sarcoma is used to describe tumors arising from the intralobular stroma of the breast (4). Pure stromal sarcomas of the breast are very rare and only few cases have been reported until date (5). Stromal sarcomas of the breast were defined in 1962 as a group of mesenchymal malignant tumors with fibrous, myxoid and adipose components excluding malignant cystosarcoma phylloides, lymphomas, and angiosarcomas (3). As these sarcomas are composed of specialized type of stroma, their appearances do not match with those arising in usual soft tissue locations. The recently reported cases of CD10 positive mammary sarcomas are examples of this phenomenon (8). Our case also showed expression of CD10 in tumor cells.

The predisposing risk factors for breast sarcoma are not clearly known. Following conditions are seen to be associated with breast sarcoma development: a) external beam radiation of the breast or chest wall, b) chronic lymphadema of the breast and arm, c) preexisting fibroadenomas, d) hereditary conditions like neurofibromatosis or Li-Fraumeni syndrome (1). 

The typical clinical presentation is a unilateral breast mass that grows in size more rapidly than an epithelial breast cancer (4). Breast sarcomas can grow very large (10). The size of these tumors may range from <1 cm to >40 cm. The skin overlying the tumor may be discolored (4). These tumors are at high risk of recurrence and are known to have poor prognosis. They tend to spread by direct local invasion or hematogenously (5). As with soft tissue sarcomas of other sites, metastases from primary breast sarcoma typically occur hematogenously involving the lungs, bone marrow and liver (4).

Early diagnosis and treatment affect survival. Delay in breast cancer diagnosis is associated with negative clinical effects (2). Nowadays imaging techniques like mammography, ultrasound and MRI are used in presumptive diagnosis of primary breast sarcoma. However, because breast sarcoma is rare, analysis of its imaging characteristics has been limited (10). Mammography is nonspecific for diagnosis as calcification is rare (4). Histopathothological analysis has a great importance in diagnosis of primary breast sarcomas along with an immunohistochemical panel that is valuable in assessment of suspected metaplastic spindle cell tumor (5). In our setting, immunohistochemistry was a major input in diagnosis.

The differential diagnosis in cases of primary soft tissue sarcomas of the breast must be considered. It includes sarcomatoid carcinoma, carcinosarcoma, fibromatosis, nodular fasciitis and fibrous histiocytoma (4). Distinction with metaplastic carcinoma is important for treatment as well as for prognosis (10). Specific morphological features (biphasic tumor with leaves like architecture and epithelial component) recognize the former and extensive sampling of the tumor can help when a stromal overgrowth is present. The latter is recognized on H&E sections by the presence of a carcinomatous component or based on cytokeratin immunopositivity of the neoplastic spindle cells (3).

The treatment for breast sarcomas is planned by a multidisciplinary team following the treatment model of sarcomas in other locations (7). However, there is still no definitive consensus regarding the treatment of primary breast sarcomas (PBS) (6). Surgery represents the only potentially curative modality (5). Mastectomy without axillary lymph node dissection is treatment of choice for primary breast sarcoma. Few selected patients may be treated with wide local excision (6). Because the axillary lymph nodes are seldom involved axillary, dissection should be avoided unless they are clinically positive nodes (10). Negative surgical margin is most important determinant of local recurrence and survival in such cases (4). Adjuvant and neoadjuvant chemotherapy and radiotherapy should be considered in high-risk cases (7). Adjuvant radiotherapy has been recommended especially for large or high-grade tumors. The role of chemotherapy, however, is unclear (10) and can be proposed to patients with the worst prognosis (6).

Overall, the prognosis of primary breast sarcoma is poor (2). The prognosis of PBS is dependent on the tumor grade, tumor size and histological type (angiosarcoma vs others) and for most patients therapy can be similar to that administered for soft tissue sarcomas of other sites (6). Tumors with size measuring less than 5 cm are associated with better outcome (10). Like in breast carcinoma, delay in its diagnosis has important clinical and treatment implications (2). In our case, patient ignorance significantly contributed to the delay. The reported 5-year survival rates for patients with primary breast soft tissue sarcomas range from 14% to 91% (4).

## Conclusion

In our case, patient delay in the treatment influenced the morbidity profile like large tumor mass with ulceration and weakened state. Although rare, one must keep in mind the possibility of sarcoma in the breast any time there are spindle cells in the sections of the tumor as breast conservation surgery can be done without axillary dissection in view of rare spread of sarcomas by lymphatics hence, early diagnosis is crucial. 

## Conflict of Interests

The authors declare that there is no Conflict of Interests. 
